# Presence of DQ2.2 Associated with DQ2.5 Increases the Risk for Celiac Disease

**DOI:** 10.1155/2016/5409653

**Published:** 2016-11-30

**Authors:** Lucas Malta Almeida, Lenora Gandolfi, Riccardo Pratesi, Rosa Harumi Uenishi, Fernanda Coutinho de Almeida, Nicole Selleski, Yanna Karla de Medeiros Nóbrega

**Affiliations:** ^1^Graduate Program in Medical Sciences, University of Brasília School of Medicine, 70.900.910 Brasília, DF, Brazil; ^2^Research Laboratory for Celiac Disease, University of Brasília School of Medicine, 70.900.910 Brasília, DF, Brazil; ^3^Graduate Program in Health Sciences, University of Brasília School of Health Sciences, 70.900.910 Brasília, DF, Brazil; ^4^Department of Pharmaceutical Sciences, University of Brasília School of Health Sciences, 70.900.910 Brasília, DF, Brazil

## Abstract

*Background.* Celiac disease (CD) is a genetically determined immune-mediated disorder in which gluten immunogenic peptides are presented to CD4 T cells by HLA-DQ2.5, DQ8, DQ2.2, and their combinations. Our aim is to establish a risk gradient for celiac disease based on HLA-DQ profile in a brazilian representative population and the relevance of DQ2.2 in celiac disease development.* Materials and Methods.* 237 celiac patients and 237 controls (both groups with 164 females and 73 males) were included. All samples were tested for the presence of predisposing HLA-DQ alleles using the PCR-SSP method. Results were considered significant when *p* < 0.05. Disease risk was expressed as 1 : *N* for each HLA-DQ category described at this study.* Results.* DQ2.5 and/or DQ8 were detected in 224 celiac patients (94.5%) and 84 controls (35.4%). Eight celiac patients (3.4%) and 38 controls (16%) disclosed only DQ2.2. Even though DQ2.2 (*β*2/*β*2 or *β*2/x) showed a low CD risk of 1 : 251 and 1 : 550, respectively, the genotype DQ2.5/DQ2.2 (*β*2/*β*2) showed high CD risk of 1 : 10 (*p* < 0.0001). The disease risk gradient ranged from 1 : 3014 to 1 : 7.* Conclusion.* Our study allowed the determination of a risk gradient for celiac disease development in at-risk population, showing that DQ2.2 variant was relevant when associated with DQ2.5.

## 1. Introduction

Celiac disease (CD) is a genetically determined immune-mediated disorder, in which individuals carrying specific HLA haplotypes (DQ2 and/or DQ8) mount an immunologic response to the ingestion of gluten that leads to a broad range of clinical signs and symptoms. Gastrointestinal disorders are the most common manifestations and include chronic diarrhea, abdominal distention, and nutrients malabsorption. However, extraintestinal manifestations are also frequent and include numerous conditions such as dermatitis herpetiformis, anemia, dental enamel hypoplasia, osteoporosis, and neurologic problems [1].

CD activity is characterized by the production of IgA anti-endomysial antibody (IgA-EmA) and IgA anti-transglutaminase antibody (IgA-tTG), which are good markers of the active phase of the disease and are usually used as a first step in its diagnosis. In most cases, a definitive diagnosis requires a jejunal biopsy showing typical histologic abnormalities such as villous atrophy, crypt hyperplasia, and lymphocytic infiltration [2].

In the general population of Europe, United States, and countries predominantly populated by individuals of European origin, the prevalence of CD is approximately 1%. In Brazil, several prevalence studies performed to date revealed significant differences around the country, probably consequent to genetic and environmental factors and possibly also due to interlaboratory assay variability [3–7]. Although multicenter epidemiological studies that could yield reliable information on CD prevalence in Brazil are still lacking, the existing reports suggest that the disease prevalence in this country is similar to the general prevalence found in other areas of the world [2, 4, 5, 7].

Virtually all CD patients carry the alleles that code for DQ2 and/or DQ8 molecules or at least for one chain of the DQ2 heterodimer, normally *β* chain, encoded by DQB1∗02 allele. The occurrence of CD in the absence of these at-risk DQ factors is extremely rare [8]. The presence of these molecules does not predict with accuracy that CD will develop, since they are present in 25 to 50% of the general population, although the vast majority of these individuals will never develop the disease [9].

Consequently, in view of the nearly 100% negative predictive value, the HLA typing has been used as a screening tool in high-risk population such as carriers of type 1 diabetes, Down syndrome, or Turner syndrome [10]. HLA typing has also been used as a prognostic factor of the disease severity [11, 12] and sex distribution [13] and as an accessory element in the diagnosis of difficult cases [14]. Finally, the HLA-DQ typing to determine the future risk of CD has been extensively discussed, although its practical usage remains not clinically defined. Genetic testing of individuals could eliminate more than 60% of the population considered to have a low CD-risk (DQ2 or DQ8 negative) from future antibody testing, and the identification of high-risk individuals would allow a prospective screening, enabling an early therapeutic intervention [15].

As far as we know, no previous study has focused on the frequency of CD predisposing HLA genotypes in affected and nonaffected individuals in a Brazilian population. Consequently, our aim in the present study is to determine the frequency of CD predisposing DQ genotypes in celiac and nonceliac subjects and establish a CD-risk gradient focusing on the prevalence of DQ2.2, in Brazil.

## 2. Material and Methods

### 2.1. Celiac Patients and Ethics Committee

This retrospective study included celiac patients followed during the period of 2006 to 2014 at the Celiac Disease Outpatient Clinic of the Brasilia University Hospital. The diagnosis was achieved according to the criteria of the European Society of Pediatric Gastroenterology and Nutrition (ESPGAN) [16] and the revised guidelines of the European Society of Pediatric Gastroenterology, Hepatology and Nutrition (ESPGHAN) [17]. The study was approved by the School of Health Sciences Ethic Committee in Human Research (project number 070/06) and is in accordance with the latest Declaration of Helsinki. Written informed consent for participation was obtained from all participants prior to their enrollment in the study, including consent to publish.

### 2.2. Patients and Controls

237 serologically and biopsy confirmed celiac patients were included in the study (164 females and 73 males, age range: 1 to 75, mean age 21.5 ± 16.2 years at sample collection). Control group included 237 unrelated, gender and age matched, healthy individuals (164 women and 73 males, age range: 1 to 75 years, mean age 18.5 ± 16.14 years at sample collection), without history of autoimmune diseases and from the same geographic area and socioeconomic stratum as the celiac patients. All controls underwent serologic tests: IgA anti-transglutaminase antibodies (QUANTA Lite™ h-tTG IgA ELISA, INOVA Diagnostic, Inc., San Diego, CA, USA) and IgA anti-endomysium antibodies (NOVA Lite® Monkey Oesophagus IFA Kit, INOVA Diagnostic, Inc., San Diego, CA, USA) and disclosed negative results.

### 2.3. HLA Alleles Genotyping

Whole blood was obtained from celiac patients and controls in EDTA containing tubes following the H3-A6 criteria of Clinical and Laboratory Standards Institute (CLSI). DNA extraction was performed using commercial kit (Illustrat™ Blood Genomic Prep Mini Kit, GE Healthcare, Buckinghamshire, UK), and quantified with a NanoVue spectrophotometer (GE Healthcare, Buckinghamshire, UK). The final concentration of DNA was adjusted to 20 ng/*µ*L.

The amplification of the alleles DQA1∗05, DQA1∗02:01, DQB1∗02, DQA1∗03, and DQB1∗03:02 was performed utilizing the commercial DQ-CD Typing Plus Kit (BioDiagene®, Palermo, Italy), which also allows the detection of homozygosis of the DQB1∗02 allele.

### 2.4. Statistical Analysis

Celiac patients and controls were categorized according to their HLA-DQ profile. Fisher's exact and *χ* ^2^ tests were applied to compare the frequencies of predisposing HLA-DQ alleles between celiac patients and controls. Results were considered significant when *p* < 0.05. The risk assessment calculations was based on the study by Megiorni et al. [18], in which the degree of risk was represented by 1 : *N*, where *N* was the number of healthy individuals among which one patient is present. The analysis was performed considering a presumed prevalence of 1 : 100 in the general Brazilian population. For each HLA-DQ category, *N* was calculated as a percentage of controls with a distinct HLA-DQ multiplied by 100 and divided by the percentage of patients with the same DQ typing.

### 2.5. Nomenclature

The DQ2.5 HLA heterodimer is encoded by the allelic variants DQA1∗05 and DQB1∗02. These two variants may be present either in* cis* (haplotype DR3) or in* trans* conformation (haplotype DR5/DR7). The HLA heterodimer DQ2.2 is encoded by the alleles DQA1∗02:01 and DQB1∗02:02, which may be found in both heterozygosis (haplotype DR7/X) and homozygosis (haplotype DR7/DR7).

Considering that previous studies have shown that the dose-dependent effect of the allele DQB1∗02 significantly increases the degree of risk, we classified as *β*2/x those individuals with only one copy of the DQB1∗02 allele (heterozygosis) and as *β*2/*β*2 those with two copies of the same allele (homozygosis). Individuals classified as *β*2/x may show either DR3/X, DR5/DR7, X/DR7, or DR7/DR4 haplotypes. Individuals pertaining to the group *β*2/*β*2 may show haplotypes DR3/DR3 or DR3/DR7 and DR7/DR7.

The HLA-DQ8 molecule is encoded by allelic variants DQA1∗03 and DQB1∗03:02 which are in linkage disequilibrium and consequently are commonly present in* cis-*conformation in the DR4 haplotype. This haplotype can be found either in association with DQ2.5 (DR3/DR4), DQ2.2 (DR7/DR4) or with DQA1∗05 (*α*5/DR4).

The *α*5 individuals carry the allele DQA1∗05 and *α*8 individuals carry the allele DQA1∗03, without any other CD predisposing allele. Individuals without any CD predisposing alleles were designed as absent. “X” refers to undetermined haplotypes and “x” to undetermined alleles.

## 3. Results

The presence of HLA-DQ2.5/DQ8/DQ2.2 genotypes was categorized according to the presence of CD predisposing HLA alleles (Table 1). The HLA-DQ2.5 and/or DQ8 were detected in 224 celiac patients (94.5%) and in 84 (35.4%) controls. Eight celiac patients (3.4%) and 38 controls (16%) disclosed the HLA-DQ2.2 either in homozygosis or in heterozygosis.

The genotype DQ2.5/DQ2.5, which confers an increased risk of developing CD, was found in 15 (6.33%) celiac patients and in a single control (0.42%), while the genotype DQ2.5/DQ2.2 was found in 60 (25.31%) celiac patients and 6 controls (2.53%).

The DQ2.5 genotype, when not associated with DQ8, was present in 178 (75.1%) celiac patients and 38 (16%) controls. Ten celiac patients (4.2%) and 36 controls (15.2%) disclosed only DQ8 alone, unaccompanied by DQ2.5 nor DQ2.2.

Celiac patients and controls, who did not disclose any of the risk variants (DQ2.5, DQ2.2, or DQ8), were explored for the presence of alleles that could confer low risk for the development of CD. The frequency and absence of these alleles, in celiac patients and controls, are shown in Table S1 (see Supplementary Material available online at http://dx.doi.org/10.1155/2016/5409653).

**Figure 1:**
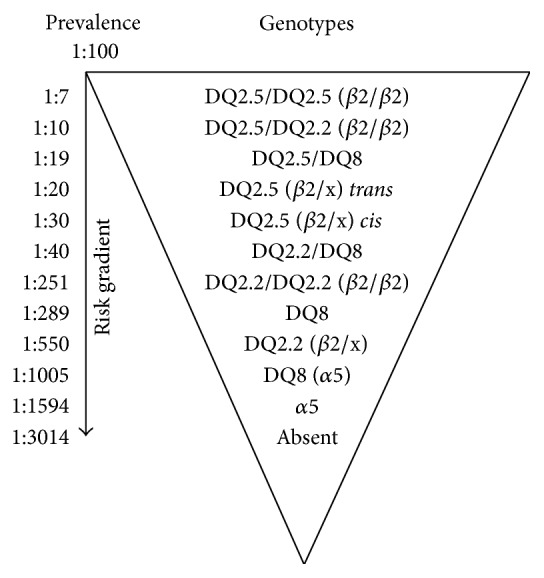
Risk gradient according to HLA haplotype combinations, considering a disease prevalence of 1 : 100. *β*2 refers to DQB1∗02; *α*5 refers to DQA1∗05; “x” denotes corresponding allele not determined; individuals with only one copy of the DQB1∗02 allele were classified as *β*2/x (heterozygosis); individuals with two copies of the DQB1∗02 allele were classified as *β*2/*β*2 (homozygosis);* trans* and* cis* indicate, respectively, *α*5 and *β*2 position in different or in the same chromosome 6 pair.

The comparison between the frequency of CD HLA predisposing alleles in celiac patients and controls was used to establish a disease risk gradient that, in our sample, ranged from 1 : 7 to 1 : 3014 (Figure 1).

The highest risks findings (1 : 7) were for the genotype DQ2.5/DQ2.5 (*β*2/*β*2) positive subjects (*p* = 0.0004, OR 15.95 95%, CI 2.08–121.8) and DQ2.5/DQ2.2 (*β*2/*β*2) (1 : 10) with *p* < 0.0001, OR 13.05 95%, CI 5.51–30.9.

The risk related to the concomitant presence of DQ2.5 and DQ8 was 1 : 19 (*p* = 0.0007; OR 5.66 95% CI 1.91 to 16.77). The category DQ2.5 (*β*2/x) in either* trans-* (DR5/DR7) or* cis*-configuration (DR3/X) disclosed different risk values 1 : 20 (*p* = 0.0002; OR 5.47 95% CI 2.05 to 14.55) and 1 : 30 (*p* < 0.0001; 4.35 OR 95% CI 2.59 to 7.30), respectively.

The presence of DQ2.2/DQ8 was associated with a risk of 1 : 40 (*p* = 0.0718; OR 2.60 95% CI 0.991 to 6.826), while the presence of DQ2.2/DQ2.2 (*β*2/*β*2) was associated with a risk of 1 : 251 (*p* = 0.4496; OR 0.39 95% CI 0.085 to 2.06). The DQ2.2 (*β*2/x) showed a risk of 1 : 550 (*p* < 0.0001; OR 0.16 95% CI 0.08–0.39), while DQ8 without any other CD predisposing allele disclosed a risk of 1 : 289 (*p* = 0.0043; OR 0.32 95%, CI 0.146 to 0.699).

The association of DQ8 and *α*5 disclosed a significant decrease in disease risk of 1 : 1005 (*p* = 0.0108; OR 0.096 95% 0.0122–0.758), when compared to the presence of DQ8 alone. Finally, the exclusive presence of *α*5 showed a risk of 1 : 1594 (*p* < 0.0001; OR 0.050 95% CI 0.015–0.165), while the complete absence of any CD-HLA-DQ predisposing alleles was associated with a risk of 1 : 3014 (*p* < 0.0001; OR 0.024 95% CI 0.006–0.101). The risk for the presence of *α*8 was undetermined.

## 4. Discussion

The onset of CD is strongly associated with the presence of HLA-DQ2 and DQ8, which are considered to account for up to 40% of the genetic risk for the disease development. Other non-HLA genes are involved in CD susceptibility, although their genetic contribution to CD is weak and is not generally considered in the calculation of the disease risk [19, 20]. Consequently, CD risk calculation, based on the presence of these HLA alleles, will allow a practical evaluation of the need for subsequent periodic tests on subjects included in the at-risk group. On the other hand, in view of the high negative predictive value of their absence, the need of further serologic testing is practically excluded in negative subjects.

Data obtained in the present study regarding the distribution of haplotypes containing DQ2.5/DQ8/DQ2.2 and their corresponding alleles, in a representative sample of Brazilian celiac patients and of presumably healthy controls, were used to calculate the risk gradient for future development of celiac disease in our population.

The Brazilian population has a high degree of genetic heterogeneity resulting from more than 500 years of interbreeding among three main ethnicities: Europeans, Amerindians, and Africans. In addition, during the last two centuries, successive migratory waves of Italians, Spaniards, Germans, Japanese, Lebanese, and Syrian further increased our population racial miscegenation. The analysis of ancestry informative markers of a representative sample of the Brazilian population disclosed a major contribution of Europeans (0.771%), followed by Africans (0.143%), and Amerindians (0.085%) [21]. The current population of Brasilia can be considered representative of the Brazilian population, since, during more than fifty years from its foundation, this city, presently with more than 2,500,000 inhabitants [22], has hosted people from all over the country.

Despite the great ethnical diversity of the distinct macroregions of Brazil, the frequency of different haplotypes in Brazilian celiac patients is relatively stable. Previous studies performed in small series of celiac patients revealed that HLA-DQ2.5 and/or DQ8 were present in 93.1% of celiac patients in the Northeastern region of Brazil, where the miscegenation with Africans and Amerindians is more intense [23]. In the Southern region, where Caucasian ancestry predominates, the frequency of 91.1% was found [24]. In this study, conducted in the Midwestern region of the country, the frequency of these genotypes was 94.5%. It is of interest that these frequencies are very similar to those found in Europe, where over 90% of the celiac patients carry the DQ2 heterodimer, the remaining being mostly characterized by the presence of DQ8 [8]. Only a small number of celiacs carry neither the DQ2 nor the DQ8 genotypes, although several have been reported to have just one chain of the DQ2 heterodimer [8].

Additionally, in our study, when considering the presence of the DQ2.5 variant without association with DQ8, the frequency found in celiac patients was 75.1%, while in previous studies performed in the Southern and Northeastern regions of Brazil was 65.3% [24] and 68.5%, respectively [23]. The presence of DQ8 alone (i.e., without the concomitant presence of DQ2.5) among our celiac patients was 10.5%. Comparatively, the frequency in the previously cited regions was, respectively, 11.9% and 17.8% [23, 24].

Interestingly, when comparing celiac patients and controls for the presence of DQ8, alone or in combination with other genotypes or alleles (Table 1), the same frequency of 19.4% was detected in both groups. The only differences between groups were the DQ8 allelic combinations and their related risks. The association of DQ8 with DQB1∗02 (DQ2.5/DQ8 or DQ2.2/DQ8) is predominantly observed in celiac patients and in a minor degree in controls. The presence of this association increases 7 to 15 times the risk of developing the disease compared to DQ8 alone. These data are in agreement with the fact that the presence of DQB1∗02 is associated with an increased risk of CD due to its higher antigenic repertoire, a fact which does not occur with an uncombined DQ8 [8, 18, 25, 26].

The low frequency of DQ2.2 (*β*2/x and *β*2/*β*2) (Table 1) found among celiac patients (*n* = 8, 3.4%) when compared to controls (*n* = 38, 16%) shows that although this variant is a predisposing factor for CD, it has a minor capacity to trigger an autoimmune process. This fact is in accordance with low binding stability of DQ2.2 heterodimer with gluten antigens decreasing the inflammatory response [27, 28]. However, its genotyping is recommended in at-risk groups; since the genotype DQ2.2 is present in association with DQ2.5 and DQ8, it becomes a major risk factor significantly increasing the risk for CD onset [29].

In the present study, the highest risk for CD was associated with the presence of the HLA genotypes DQ2.5/DQ2.5 (*β*2/*β*2), DQ2.5/DQ2.2 (*β*2/*β*2), and DQ2.5/DQ8 that disclosed a risk of 1 : 7, 1 : 10, and 1 : 19, respectively. In comparison, in the Italian population the risk for CD was due to the presence of the HLA genotypes DQ2.5/DQ2.5 (*β*2/*β*2) and DQ2.5/DQ2.2 (*β*2/*β*2) and DQ2.5/DQ8, which were associated with a respective risk of 1 : 10 and 1 : 7 [18].

In our study, the DQ2.5 heterodimer disclosed a risk of 1 : 20 (in* trans*) and 1 : 30 (in* cis*), showing statistically significant differences. However, when* cis-* and* trans-*conformations of DQ2.5 were considered together, the resulting risk was 1 : 30 (data not shown), similar to the risk found in the literature [18, 30].

The combination DQ8/*α*5 showed 3.5 times decreased risk of developing CD than that reported for isolated DQ8 (Figure 1). Similar relationship was found in Southern Spain [30]. Both patients and controls with this combination disclosed a DR5/DR4 haplotype configuration. It could be possible that the* trans*-conformations (DQA1∗05/DQB1∗03:02 and DQA1∗03/DQB1∗03:01) lead to the production of not very efficient heterodimers regarding to the presentation of some major immunogenic gluten peptides, since there is a lack of evidence for these events in the current study [26]. Consequently, we suggest that the association of DQA1∗05/DQB1∗03:02 and DQA1∗03/DQB1∗03:01 reduces the binding of immunogenic epitopes of gluten and transglutaminase 2, therefore not triggering an effective immune response mediated by lymphocytes B and T.

Individuals belonging to at-risk groups that only show the *α*5 allele or do not disclose any CD predisposing HLA alleles could be excluded from periodical follow-ups in view of their extremely low risk of developing CD [8, 18] (Figure 1).

## 5. Conclusion

The present study showed, in a representative population of celiac patients and controls, the frequency of HLA-DQ variants DQ2.5/DQ2.2/DQ8, determining the probability of developing CD and establishing a risk gradient for the future onset of the disease. These data are useful for the screening of subjects included in at-risk groups for CD, discriminating subjects that are unlikely to develop the disease from those who must be monitored for a possible future development of CD. Additionally, this study demonstrated that although the HLA-DQ2.2 allele has a negligible risk factor when alone, its importance for the onset of CD will significantly increase when associated with DQ2.5.

## Supplementary Material

Celiac patients and control clinical data and complete celiac predisposing HLA-DQ alleles, including all HLA class II alleles associated with Celiac Disease, genotypes, haplotypes included in this study.

## Figures and Tables

**Table 1:** CD predisposing HLA-DQ frequencies in CD patients and controls and general genetic risk.

Genotypes	DQB1∗02 status	CD% (*n* = 237)	Control (*n* = 237)	*p*CD/control	General risk
					1 : 100
DQ2.5/DQ2.5	(*β*2/*β*2)	6.33% (15)	0.42% (1)	0.0004	1 : 7
DQ2.5/DQ2.2	(*β*2/*β*2)	25.31% (60)	2.53% (6)	<0.0001	1 : 10
DQ2.5/DQ8	(*β*2/x)	8.86% (21)	1.68% (4)	0.0007	1 : 19
DQ2.5	(*β*2/x) *trans*	10.54% (25)	2.11% (5)	0.0002	1 : 20
	(*β*2/x) *cis*	32.91% (78)	11% (26)	<0.0001	1 : 30
DQ2.2/DQ8	(*β*2/x)	6.33% (15)	2.53% (6)	0.0718	1 : 40
DQ2.2/DQ2.2	(*β*2/*β*2)	0.84% (2)	2.11% (5)	0.4496	1 : 251
DQ8	—	3.8% (9)	10.97% (26)	0.0043	1 : 289
DQ2.2	(*β*2/x)	2.53% (6)	13.92% (33)	0.0001	1 : 550
DQ8/*α*5	—	0.42% (1)	4.22% (10)	0.0108	1 : 1005
*α*5	—	1.3% (3)	20.25% (48)	<0.0001	1 : 1594
Absent	—	0.84% (2)	25.31% (60)	<0.0001	1 : 3014
*α*8	—	0.0% (0)	2.95% (7)	0.0149	ND

ND: risk not determined; CD: celiac disease; *β*2 refers to DQB1∗02; *α*5 refers to DQA1∗05; *α*8 refers to DQA1∗03:01; x denotes not determined allele; *β*2/*β*2 refers to DQB1∗02 homozygosis; *β*2/x refers to DQB1∗02 heterozygosis; *p* value.
